# Role of Stem Cell Transplantation in Multiple Myeloma

**DOI:** 10.3390/cancers13040863

**Published:** 2021-02-18

**Authors:** Srinivas Devarakonda, Yvonne Efebera, Nidhi Sharma

**Affiliations:** Division of Hematology, Department of Internal Medicine, The Ohio State University Comprehensive Cancer Center Columbus, Columbus, OH 43210, USA; srinivas.devarakonda@osumc.edu (S.D.); Nidhi.Sharma@osumc.edu (N.S.)

**Keywords:** stem cell transplantation, multiple myeloma, autologous, allogenic, maintenance

## Abstract

**Simple Summary:**

The aim of this review is to provide an overview of the current scientific evidence concerning the role of stem cell transplantation in multiple myeloma. During the past decade, several new treatment options have become available, thereby questioning the role of stem cell transplantation for the management of multiple myeloma. This review focuses on these studies, demonstrating a benefit for autologous stem cell transplantation (auto-SCT). We also reviewed maintenance post auto-SCT and utility of allogeneic stem cell transplant.

**Abstract:**

Autologous stem cell transplantation (auto-SCT) has been the standard of care in eligible newly diagnosed multiple myeloma (MM) patients. Outcomes of patients with MM have improved significantly due to the advent of several novel drugs. Upfront use of these drugs in induction therapy has significantly increased the rate and depth of responses that have translated into longer remission and survival. This has now raised a debate regarding the role and relevance of auto-SCT in the management of myeloma. However, clinical trials have confirmed the utility of auto-SCT even in the era of novel drugs. Tandem auto-SCT followed by maintenance has shown a progression-free survival (PFS) benefit in high-risk MM, and hence can be considered in young and fit patients with high-risk disease. Auto-SCT has the advantages of resetting the bone marrow microenvironment, short-lived toxicity compared to the long-term physical and financial toxicities of continued chemotherapy in the absence of SCT, very low transplant-related mortality (TRM) in high volume centers, and providing longer disease-free survival when followed by maintenance therapy. Allogeneic SCT is one potentially curative option for MM, albeit with an increased risk of death due to high TRM. Strategies to modulate the graft-versus-host disease (GVHD) while maintaining or improving the graft-versus-myeloma (GVM) effect could place allogeneic SCT back in the treatment armamentarium of MM.

## 1. Introduction

Multiple myeloma (MM) accounts for about 10% of all hematologic malignancies [1]. It is a malignant disorder in which plasma cells accumulate in the bone marrow and secrete either an entire immunoglobulin (usually IgG or IgA) and light chain (kappa or lambda) or only immunoglobulin light chains. It has been shown to arise from a benign condition monoclonal gammopathy of undetermined significance (MGUS). Often it can behave in an aggressive manner and can become resistant to most chemotherapeutic drugs. Multiple myeloma is a heterogeneous disease and hence requires treatment to be more personalized [2].

About 35 years ago, alkylating agents and corticosteroids were the most effective conventional agents for the treatment of this disease but without any major improvement in the outcomes [3]. Intravenous melphalan was then introduced at high doses to overcome drug resistance. This, however, induced severe and prolonged myelosuppression [4,5]. The functional bone marrow was restored by the infusion of autologous hematopoietic stem cells that were collected before the administration of melphalan. The Intergroupe Francophone du Myélome was the first to conduct a randomized trial showing the superiority of this approach, as compared with conventional chemotherapy [6]. Several other studies compared conventional chemotherapy with ASCT, showing a significant impact on progression free survival, but failed to show a significant improvement in overall survival [7].

The efficacy of high-dose chemotherapy is mostly related to damage to tumor-cell DNA. It has, however, been shown that agents such as thalidomide, bortezomib, and lenalidomide act not only on myeloma cells, but also on the tumor microenvironment [8]. Hence, these agents may offer another means of overcoming drug resistance. In addition, some studies have tried to evaluate the role of novel agents as part of maintenance therapy post ASCT [9,10]. An important finding from these studies was a significant increase in complete remission with ASCT, results that translated into significantly prolonged progression-free survival (PFS) and overall survival (OS). Although transplantation improved the response rate and progression-free survival, there was no overall survival benefit in most of the trials [11]. This has been thought to be partly related to the patient selection criteria and different conditioning therapy in the trials. In addition, significant benefit with transplantation at relapse in patients who were initially treated with conventional chemotherapy raised the question of early versus delayed ASCT [12]. In addition, progress has been made on the role of allogeneic stem cell transplantation (allo-SCT) in relapsed/refractory settings in the era of combination of novel agents. Advances in the management of toxicity and improved GVHD prophylaxis have allowed allo-SCT to be used in the treatment of relapsed and refractory multiple myeloma (RRMM), especially in patients who have already undergone prior ASCT. This review discusses the efficacy of stem cell transplantation in the treatment of multiple myeloma and its role in the era of novel drugs and treatment modalities.

## 2. Patient Eligibility for Autologous SCT in MM

### 2.1. Patient Related Factors

ASCT is offered as consolidation therapy to young patients and eligible elderly (older than 65 years) patients with newly diagnosed MM (NDMM). There is no age cut-off for ASCT eligibility, at least in the United States. In a study by Wildes et al. [13], after adjusting for performance status, disease stage, and comorbidities, patients older than 65 years who underwent ASCT for MM had prolonged survival compared to those who did not. In a single center retrospective analysis of patients older than 70 years who received ASCT, outcomes were comparable to those seen in younger patients [14]. The peak incidence of MM in older age along with increase in life expectancy and longer disease related survival with the use of novel drugs has created an increased number of older adults for whom ASCT is an option. As such, MM is one of the most common indications of ASCT. In the last decade, 44% of the ASCT recipients were at least 60 years of age according to data from CIBMTR, which is an encouraging trend [15]. Even on the European side, ASCT for MM in patients 70 years and older has increased from 1.1% to 3% between 2006 and 2010 [16]. The significance of biological age in the outcomes of ASCT in MM, especially increased non-relapse mortality (NRM) has been well studied. Hayden et al. [16] reported increasing day +100 death with advancing age in ASCT recipients in MM (2.4% in patients ≥70 years compared with 1.2% in patients 40–49 years and 0.7% in those younger than 40 years of age). However, due to the heterogeneity of the effects of ageing, it is difficult to assess eligibility for ASCT solely based on biological age. Functional or physiological age rather than chronological or biological age should be the determining factor for ASCT eligibility in MM. The former is a reflection of patient’s performance status along with their health reserve that correlates better with how well they tolerate the stem cell transplantation. The next important determinant of transplant outcomes that has been well studied and recognized is comorbidity. The Hematopoietic Cell Transplantation Comorbidity Index (HCT-CI) is one such index that is based on the presence of pre-SCT comorbidities and organ dysfunction, and has been shown to correlate well with survival and non-relapse mortality of patients after transplant. Day+100 mortality post ASCT was 3% for patients with an HCT-CI score of ≥3 [17]. Elderly patients need a thorough evaluation of their overall health status and functional reserve and not just a brief evaluation in the clinic. Geriatric assessment is a comprehensive evaluation of the functional capacity of elderly patients, and it is a summative assessment of several domains of health including comorbidities, physical performance (based on both patient report and objective evaluation), nutritional status, cognitive and psychological capacities that determine disease outcomes [18,19]. Lack of social support and polypharmacy are other major issues that influence the outcomes of ASCT in elderly patients and need to be addressed during transplant evaluation. Consideration of functional age and comprehensive geriatric evaluation will help in more accurate assessment of transplant eligibility and post-transplant outcomes.

### 2.2. Renal Insufficiency and ASCT

Renal insufficiency (RI) is prevalent in 20–50% of the MM patients, and a portion of these patients are dialysis dependent [20]. ASCT has been the mainstay of treatment in young multiple myeloma patients with normal renal function but has been of concern for RI patients [21]. In the past, reports have shown high dose melphalan with ASCT to be effective even in myeloma patients with RI [22,23]. The majority of the previously published data suggests increased toxicity, including infections and mucositis, in patients with RI [22,24,25]. Studies evaluating the effectiveness and toxicity of high dose melphalan conditioning in patients with either normal renal function or RI showed that patients in the RI group experienced more mucositis and infections compared to patients in the normal group [22,24]. However, the decision to do ASCT or not is usually at the physician’s discretion depending on several other factors as mentioned above. Melphalan to date has been shown to be an effective conditioning regimen; thus, the question arises as to what the effective dose of melphalan in patients with RI is. Studies have reported high-dose melphalan to yield a good hematological response but poor survival outcomes compared to lower doses [24,25]. There have been conflicting reports on the most effective dose of melphalan in RI, partly due to the heterogeneity of the studies for the outcome measure (clinical response or remission status). In a recent study, Mahindra et al. showed that melphalan 200 mg/m^2^ was safe in both moderate and severe RI [23]. Moderate RI patents had improved outcomes with high doses, and importantly, a portion of dialysis patients achieved dialysis independence post ASCT. Thus, the escalation of melphalan dose may result in better response rates in patients with mild to moderate RI. Lower doses at 140 mg/m^2^, however, have been seen to have lower associated mortality and be beneficial for renal recovery [24,25,26,27]. Unlike other hematological malignancies, MM patients on dialysis are not excluded from having ASCT. In these patients, lower doses (140 mg/m^2^) are routinely used.

## 3. Induction Therapy—An Optimal Approach

Immunomodulatory drugs and proteasome inhibitors are used along with steroids as backbone for induction therapy to reduce tumor burden and improve quality of life. This has led to improved patient outcomes, but there is ongoing research to improve upon this regimen. Current induction regimens consist of novel therapies, which include immune modulators (Imids) and proteasome inhibitors (PIs), such as bortezomib (V), lenalidomide (R), and dexamethasone (D) (VRD); V/thalidomide(T)/D (VTD); V/Cyclophosphamide (C) (VCD); and carfilzomib (K)/R/D (KRD). A direct comparison of thalidomide and lenalidomide as part of induction therapy was reported in the myeloma XI trial. Myeloma XI was a randomized trial that showed the superiority of lenalidomide over thalidomide in combination with cyclophosphamide in NDMM with 60% of patients achieving at least VGPR after induction with CRD. The results are comparable to other novel triple agent regimens [28]. Several studies have established the superiority of a triplet combination of VRD and VTD over doublet regimens [29,30,31]. In addition, a combination of an IMiD and PI was found to be superior to VCD [32]. Recently, combination of Dara-VTD and Dara-VRD have made it into the induction regimens, as they have been shown to confer deeper responses compared to triplet regimens [33,34].

It is widely known that ASCT is usually performed in patients who are responsive to chemotherapy, but there has been no consensus regarding the ideal pre-SCT response needed for ASCT that would improve survival. There have been conflicting reports regarding the effect of post-induction disease response on the benefit obtained from upfront ASCT. Studies have shown that the attainment of a deep response prior to ASCT was associated with a survival advantage [35], and failure to achieve at least a PR after induction therapy with novel agents was associated with an inferior OS and PFS [36]. A study by a Spanish myeloma group showed that even patients with stable disease had outcomes comparable to those with chemo sensitive disease, but patients with progressive disease had no benefit from ASCT [37]. A multicenter retrospective study showed that salvage therapy for patients with less than partial response to induction therapy including novel agents improved pre-SCT disease response but did not alter the post-SCT outcomes such as survival [38]. In the era of novel drugs, attainment of at least partial response prior to ASCT rather than fixed number of cycles should be the standard practice.

## 4. Conditioning Chemotherapy

T.J. McElwain and R.L. Powles hypothesized that using high doses of an effective agent such as melphalan or cyclophosphamide would lead to increased proportion of cell death and overcoming drug resistance of tumors. They reported the first successful treatment of nine patients with multiple myeloma, including one patient with plasma cell leukemia after conditioning with intravenous high dose melphalan (140 mg/m^2^) followed by infusion of bone marrow graft in 1983 [4]. This was confirmed later in another series of patients by Selby et al. [39]. Barlogie and colleagues showed that the myelotoxicity of melphalan can be effectively managed with autologous stem cell rescue [40]. This paved the way for the routine use of ASCT in MM.

Several drugs have been tested in combination with high dose melphalan as conditioning chemotherapy to improve the ASCT outcomes including busulfan, bendamustine, arsenic trioxide, ascorbic acid, bortezomib, and lenalidomide. None fared better than melphalan alone [41,42,43,44]. Attempts are being made for better outcomes by supplementing melphalan with other agents. In one such study, a phase III trial comparing high dose melphalan (200 mg/m^2^) to bu-mel (busulfan i.v. 130 mg/m^2^ daily for four days followed by two daily doses of melphalan at 70 mg/m^2^) showed significantly prolonged progression-free survival (PFS) with bu-mel but no difference in overall survival (OS). However, the incidence of mucositis, transaminitis, and febrile neutropenia was significantly higher with the addition of busulfan [45]. For relapsed patients, bendamustine, an alkylating agent, has been tested in combination with high-dose melphalan as conditioning therapy for second transplant in MM patients. Addition of bendamustine resulted in deeper responses and was relatively safe as conditioning therapy for either tandem transplant in NDMM or as a second transplant in relapsed MM patients [46,47,48]. Novel therapies such as bortezomib and lenalidomide have also been tested as conditioning therapies in SCT in MM. In the Intergroupe Francophone du Myeloma (IFM) 2014-02 study, addition of bortezomib to high-dose melphalan conditioning did not improve response rates, PFS, or OS in NDMM [49]. A phase 1 trial of high-dose lenalidomide with melphalan was well tolerated, and maximum tolerated dose was not reached at 350 mg/day [50]. ASCT with total body irradiation (TBI) and melphalan (140 mg/m^2^) conditioning was compared with a conventional therapy regimen consisting of vincristine, carmustine, melphalan, cyclophosphamide, and prednisone in the phase 3, intergroup SWOG 9321 study [51]. This study allowed older patients up to 70 years of age to be enrolled. At a median follow-up of 76 months, there was no difference in the response rates or survival outcomes between the groups. Inclusion of TBI in conditioning therapy has fallen out of use now due to no proven benefit. Despite attempts for intensification of conditioning regimens, melphalan 200 mg/m^2^ remains the standard conditioning chemotherapy for ASCT in MM.

### 4.1. Evidence for the Role of SCT as Consolidation Therapy

High dose chemotherapy with autologous stem cell transplantation has been an integral part of management of young and fit elderly patients with NDMM for the last three decades. The IFM group conducted one of the earliest prospective trials in patients with NDMM, comparing the efficacy of high-dose chemotherapy followed by ASCT to conventional chemotherapy [6]. After four to six alternating cycles of vincristine, melphalan, cyclophosphamide, prednisone (VMCP) and carmustine, vincristine, adriamycin, and prednisone (BVAP), patients with a WHO performance status of less than 3 and serum creatinine less than 1.7 mg/dL received conditioning with melphalan (140 mg/m^2^) and total body irradiation with 8 Gy, followed by stem cell infusion, followed by maintenance with recombinant interferon alpha after hematologic reconstitution. Patients in the non-transplant arm received 18 cycles of chemotherapy with recombinant interferon-alpha given from cycle 9 until relapse. Patients in the ASCT arm had a higher probability of five-year event-free survival (EFS) (28% versus 12%, *p* = 0.01) as well as five-year overall survival (OS) (52% versus 12%, *p* = 0.03). These results were similar to those seen in the Southwest Oncology Group (SWOG) study done by Barlogie et al. comparing ASCT following “total therapy” with standard SWOG chemotherapy [51]. In 2003, the randomized study by the MRC group in patients younger than 65 years with NDMM showed a survival benefit of approximately one year with ASCT compared with conventional chemotherapy [52].

Several studies have been done in the last decade to investigate the role of ASCT in the era of novel drugs such as the proteasome inhibitors and immunomodulators. A study by the GIMEMA group randomized patients with NDMM to tandem transplant versus six cycles of MPR (melphalan, prednisone, lenalidomide) following Rd (lenalidomide, dexamethasone) induction, with further randomization of each arm to maintenance with lenalidomide versus placebo. Both PFS and OS were superior in the ASCT group [53]. In another study by the same investigators, Rd induction was followed by consolidation with either ASCT or six cycles of CRD (cyclophosphamide, Revlimid, and dexamethasone), showing similar outcomes of improved PFS and OS with ASCT [54]. It is of note that in both the above studies, patients older than 65 years were excluded (Table 1).

Given the increase in the rate and depth of responses with novel drug combinations of both a PI and an IMiD that has become the standard of care for NDMM, the IFM/Dana-Farber Cancer Institute (DFCI) group conducted a prospective, randomized study to explore the role and timing of ASCT in MM. In this study, patients with NDMM up to 65 years of age received induction with three cycles of VRd followed by consolidation with either five cycles of VRd or ASCT followed by three additional cycles of VRd. Both the arms received lenalidomide maintenance until progression (in the DFCI US study) or for one year (in the IFM study). Patients in the chemotherapy only arm received ASCT at first relapse. While the DFCI study is still awaiting final outcomes, the IFM study showed that upfront ASCT in patients with NDMM resulted in significantly longer PFS compared to those without. The benefit was seen across all stages and cytogenetic risk groups [55]. However, there was minimal overall survival benefit seen in the transplant group. This landmark study answered several questions regarding ASCT in MM. First, this study showed the relevance of ASCT in the management of myeloma even in the era of novel drugs. Second, it established the role of ASCT with a significant improvement in PFS as well as MRD negativity in transplant recipients, even with induction and post-SCT consolidation using novel drugs. Third, there was no survival difference between patients who received upfront versus delayed transplant, allowing patients the choice of delaying transplant until disease relapse. Lack of overall survival benefit with ASCT in this study might have been due to shorter follow-up and high percentage of patient crossover to the transplant arm. The results of the DFCI study will give us added information on OS with longer maintenance therapy. However, similar results were shown in the study by the European Myeloma Network (EMN02/HO95) in which patients received maintenance until disease progression, and still confirming a PFS benefit with upfront ASCT in NDMM [56]. Taking these results together, ASCT is the standard of care for eligible patients with NDMM today.

### 4.2. Tandem Stem Cell Transplantation in Myeloma

Tandem stem cell transplantation involves two planned, sequential autologous stem cell transplants within a period of 3–6 months. This has been an integral component of “total therapy” for MM [57]. Results of the trials exploring the utility of tandem transplantation have yielded mixed results. In the IFM94 study, which compared one with two successive autologous stem cell transplants following high dose chemotherapy, tandem transplantation resulted in improved EFS and OS [58]. Following induction with lenalidomide and dexamethasone, patients were randomized to MPR versus tandem ASCT in the GIMEMA RV-209 study, and both PFS and OS favored the tandem ASCT arm [53]. Gay et al. [54] in their RV-MM-EMN study compared tandem ASCT with chemotherapy and lenalidomide combination and showed significantly improved PFS. Patients underwent second planned ASCT if they achieved VGPR or better after the first transplant in this study [54]. However, two separate meta-analyses by Kumar et al. and Naumann-Winter et al. showed superior response rates with tandem ASCT but no difference in survival [59,60]. A prospective, randomized study by Cavo et al. [61] compared the outcomes of patients with NDMM who received single ASCT with melphalan 200 mg/m^2^ (Mel 200) or Mel 200 followed 3–6 months later by another ASCT with Mel 120 mg/m^2^ and busulfan. Tandem ASCT resulted in significantly higher CR and near CR, EFS, and PFS with no difference in OS [61]. Another prospective, randomized trial by the German group showed that single ASCT was non-inferior to tandem SCT with no difference in EFS or OS after a median follow-up of 11 years [62]. Long-term follow-up of BMT CTN 0702 (STaMINA) trial of post ASCT strategies (auto/auto, auto/VRd, auto/Len) in the upfront treatment of multiple myeloma showed no PFS or OS difference among the arms [63]. Using intent-to-treat (ITT) analysis, six-year PFS and OS were the same among Auto/Auto (43.9%; 73.1%), Auto/RVD (39.7%, 74.9%), and Auto/Len (40.9%, 76.4%) (*p* = 0.6; *p* = 0.8). However, in as treated analysis, six-year PFS for high-risk patients favored the tandem ASCT group (PFS of 43.7% and 32% for Auto/auto and Auto/Len, respectively (*p* = 0.03)). Given the PFS benefit for tandem ASCT, it could be considered for young and fit patients with high-risk disease (Table 2).

### 4.3. Timing of ASCT in Multiple Myeloma

The optimal timing of ASCT in MM has been a topic of debate, given the IFM 2009 trial showing similar OS between upfront and delayed transplant, along with the deep and durable responses that the novel drugs are conferring. Upfront ASCT, done within 12 months of diagnosis and in non-relapsing patients, has several advantages, both related to the patient and disease. ASCT is the best initial intervention in the management of NDMM, as it yields the best depth and durability of response, probably due to the chemosensitive nature of the disease early in the course. Incorporation of ASCT early in the management of myeloma will shorten the duration of multi-drug chemotherapy, thereby avoiding the toxicity associated with it. Given the exorbitant prices of the novel drugs, early ASCT could reduce the financial burden to the patient. ASCT has unquestionable efficacy, and it confers a complete remission in one-third of transplant recipients and a median PFS of 18–27 months even without any further therapy [64]. It is a relatively safe procedure with a transplant-related mortality of <1% in experienced centers [65]. Sub analysis of the IFM 2009 study showed improvement in several functional and symptom domains of Health Related Quality of Life (HRQoL) over time to the level of general population, in spite of the short term worsening immediately following ASCT [66]. High dose chemotherapy with stem cell rescue could help reset the bone marrow and help improve its reserve. On the other hand, delayed ASCT does not negatively affect survival. In fact, toxicities related to ASCT and risk of TRM can be avoided until later [67]. However, there are a few concerns regarding delaying the transplant to the time of first relapse. First, many patients who defer ASCT end up not receiving it due to advancing age, ineligibility due to worsening performance or comorbidities, or progressive disease refractory to therapy to name a few. In the IFM 2009 study, 23% of patients in the delayed arm never underwent ASCT due to various reasons. In a pooled analysis of two European trials (RV-MM-209 and EMN-441), only half the patients who deferred ASCT received it at the time of relapse [68]. This is significant, as patients are deprived of a treatment option that can yield deep and durable responses with one time treatment with short-lived toxicity for a few weeks in the setting of an incurable disease. In addition, PFS from a salvage transplant is half of that obtained from upfront transplant [69]. This probably reflects the chemorefractoriness of disease with progression. This argues for doing upfront ASCT as opposed to delaying it in patients with advancing age and high-risk disease. ASCT could be delayed in younger patients with standard-risk disease who prefer to defer it to the time of relapse or perhaps in patients who achieve MRD negative status.

### 4.4. ASCT as Salvage Therapy

ASCT in MM is feasible and effective even when used for the treatment of relapsed disease [70,71]. In fact, it has been demonstrated to be more effective than salvage chemotherapy with improved PFS and even OS. Salvage ASCT is often done at the time of first relapse if it was not performed in the frontline setting or for a second time in patients who had a progression-free interval of at least 18 months from the time of first transplant. In a retrospective study done by the European Blood and Marrow Transplant group (EBMT), in patients who received second and third ASCT for relapses, the median OS after the third ASCT was seven months if the relapse-free interval (RFI) was < six months, 13 months if the RFI was between six and 18 months, and 27 months if the RFI was ≥18 months (*p* < 0.001) [72]. While RFI > 18 months was a favorable prognostic factor, progressive disease and Karnofsky Performance Status score of <70 at third ASCT were adverse prognostic factors for survival in a multivariate analysis. Retrospective analyses from the Center for International Blood and Marrow Transplant Research (CIBMTR) data showed that among patients who received salvage ASCT for myeloma, those who relapsed after 36 months from the first transplant did better compared to those whose disease relapsed earlier in terms of PFS and OS [73]. Longer RFI after prior transplant has been one of the most important predictors of the success with salvage transplant in all the studies. Results from the BSBMT/UKMF MRC X relapse trial, which is the only prospective study of ASCT in the salvage setting, showed that salvage transplant conferred the most benefit when it was offered right after second line chemotherapy as consolidation rather than later in the treatment course [74]. This again could be related to the chemorefractoriness of disease with progression, worsening performance status, and comorbidities with advancing age. In view of the effectiveness of salvage ASCT as an additional option in the management of RRMM, stem cells adequate for two transplants should be collected early on in patients with chemosensitive disease. Salvage ASCT has not been adequately studied, especially in the trials utilizing novel agents in the management of relapsed disease and so has been underutilized. Prospective studies comparing salvage ASCT with novel drugs, CAR-T cell therapy, and anti BCMA therapies would shed more light on the role of this modality in the treatment landscape of relapsed disease.

### 4.5. Role of Post-SCT Maintenance

Maintenance therapy has been one of the successful strategies to delay disease progression and prolong survival by deepening and sustaining the response achieved with ASCT. Prior to the era of novel drug therapy, interferon was used as post-SCT maintenance, but due to the better side effect profile and quality of life with immunomodulators and proteasome inhibitors, interferon is not used any more. Thalidomide was used as maintenance therapy with or without steroids with conflicting reports of survival benefit [75,76,77]. Results from the large, randomized Myeloma IX trial showed improved PFS and late OS benefit with thalidomide maintenance in patients with favorable interphase fluorescent in-situ hybridization (iFISH), whereas patients with adverse iFISH had no PFS benefit with worse OS [78]. Due to significant peripheral neuropathy and sedation associated with prolonged use of thalidomide, it has been supplanted by lenalidomide in this setting due to favorable side effect profile. There have been several randomized controlled trials conducted to investigate the role of lenalidomide maintenance treatment following transplant. Palumbo et al. from the GIMEMA group conducted a randomized, phase 3 study (GIMEMA-RV-209) comparing lenalidomide maintenance following transplant with no maintenance in patients with NDMM. Patients in the maintenance arm received lenalidomide for a mean duration of 35 months. Lenalidomide maintenance prolonged median PFS only (41.9 months vs. 21.6 months; hazard ratio for progression or death, 0.47; 95% CI, 0.33 to 0.65; *p* < 0.001), with no significant difference in three-year overall survival (88.0% vs. 79.2%; hazard ratio for death, 0.64; 95% CI, 0.36 to 1.15; *p* = 0.14) [53]. The IFM 2005-02 study was another phase 3, placebo-controlled trial that explored the efficacy of lenalidomide maintenance post-ASCT in NDMM patients younger than 65 years. Treatment was stopped in the maintenance arm after observing an increased number of second primary malignancies (SPM) in the lenalidomide arm. The minimum treatment duration with lenalidomide was 27 months. Lenalidomide maintenance therapy improved median PFS (41 months, vs. 23 months with placebo; hazard ratio, 0.50; *p* < 0.001) with no difference in OS [79]. The CALGB group from the United States conducted a similar trial in patients younger than 71 years to receive post-ASCT maintenance with lenalidomide or placebo. With a mean duration of treatment with lenalidomide of 30 months, both PFS and OS favored the treatment arm [80]. However, it was associated with increased toxicity, and SPMs in the lenalidomide arm was 8% vs. 4% in the placebo arm.

Given the conflicting reports on the OS benefit from maintenance therapy from these three trials, a meta-analysis of these studies was conducted by McCarthy et al., which confirmed the PFS benefit and demonstrated a significant OS benefit with lenalidomide maintenance after ASCT in patients with NDMM [81]. Lenalidomide maintenance post ASCT improved PFS in all subgroups including high-risk cytogenetics. OS benefit was not seen in patients with high-risk cytogenetics, renal disease, and high LDH. However, data on cytogenetics were not available for the majority of the patients. Increased risk of SPMs with lenalidomide was persistent even in this meta-analysis (rates of hematologic and solid tumor SPM before progressive disease were 5.3% and 5.8%, respectively, at a median follow-up of 79.5 months). It is to be noted, however, that the risk of developing progressive disease was higher than developing an invasive SPM in both the treatment and placebo groups. In addition, the PFS benefit was 52% with lenalidomide maintenance compared to a 5% risk of developing SPM. MRC XI trial later demonstrated a similar PFS benefit with post-ASCT lenalidomide maintenance compared with placebo (HR, 0.47; 95% CI, 0.38 to 0.60). This benefit was seen across all subgroups including high-risk cytogenetics [28].

Bortezomib is often used as maintenance therapy in the United States, extrapolating the PFS benefit with its use in patients with high-risk cytogenetics in the HOVON 95/GMMG-HD4 study [82]. Patients in this study were randomized to VAD (vincristine, adriamycin, dexamethasone) or PAD (bortezomib, adriamycin, dexamethasone) induction prior to single or tandem ASCT. Following transplant, patients on VAD arm received maintenance with thalidomide, while those on PAD arm received bortezomib for two years. Although the study design of this trial did not allow direct comparison between the two maintenance arms, PFS and OS were better in the bortezomib arm. No SPMs were seen with the use of bortezomib for maintenance, but peripheral neuropathy can be a potential issue with its prolonged use.

A recently published retrospective analysis comparing maintenance therapy with lenalidomide and bortezomib after upfront ASCT from two subsequent GMMG phase 3 trials showed a significant PFS benefit with lenalidomide maintenance with no difference in OS between the two groups [83].

Ixazomib, an oral proteasome inhibitor, reduced the risk of progression or death by 28% compared to placebo in the post-ASCT maintenance setting in the TOURMALINE-MM3 study [84]. Toxicity profile was favorable, and there were no increased SPMs seen with the use of ixazomib compared with placebo. Ixazomib is another oral option for post-ASCT maintenance in patients who cannot tolerate lenalidomide due to intolerance or toxicity. Early results from a large phase 2 multicenter, randomized study comparing ixazomib and lenalidomide after ASCT and consolidation with ixazomib, lenalidomide, and dexamethasone showed that more patients on the ixazomib arm discontinued treatment due to progression (30% versus 18%, respectively). Hematological and non-hematological toxicities, dose reductions, and treatment discontinuations due to toxicity were all more frequent in the lenalidomide maintenance arm [85].

In the STaMINA trial, analysis at 38 months showed that patients who continued post-ASCT maintenance beyond three years had better PFS than those who discontinued it earlier (79.5% vs. 61% at 6yr; HR = 1.91, *p* = 0.0004) with no difference in OS [63]. In view of this data, indefinite maintenance with lenalidomide is the standard of care right now. Trials are under way using response-adapted treatment strategy with minimal residual disease (MRD) status to determine the duration and intensity of maintenance therapy [86] (Table 3).

### 4.6. Allogeneic Stem Cell Transplantation in MM

Despite the improvement in response, a vast number of patients relapse, and treatment of relapse remains a major challenge. ASCT and salvage therapies at the time of relapse have led to improvement in the survival of patients with MM [87]. Nevertheless, as MM has become a chronic disease with a longer succession of remissions and relapses, finding effective treatment is critical for prolonging PFS/OS. The options include retreatment with previous regimens, and/or moving on to novel drugs or therapies. However, despite this, patients become resistant to chemotherapy and have progressive MM. Allo-SCT is a reasonable upfront salvage option in these patients and offers a potential cure. Benefit has been found to be highest when this modality is used earlier in the disease course and when used as a strategy for consolidation of remission induced by salvage therapy [88].

The earliest large retrospective, comparative study of patients who received myeloablative allo-SCT compared to those who received ASCT was reported in 1996 by EBMT. OS was inferior in the allo-SCT group due to higher transplant-related mortality (TRM), in spite of lower relapse rate [89]. Another large pooled analysis of 56,000 patients from EBMT in 2009, where patients were scored based on disease and donor characteristics, showed that patients with MM and leukemia with similar risk scores had comparable mortality rate post allo-SCT [90]. Several prospective trials investigating the role of allo-SCT in upfront treatment of MM showed encouraging results with improved response rates and PFS but with increased toxicity compared to ASCT [91,92]. In view of the increased morbidity and mortality associated with the myeloablative conditioning, there has been a lot of interest in reduced-intensity conditioning (RIC) for allo-SCT in MM, since it reduces the transplant-related mortality (TRM) significantly while preserving the GVM effect [93,94,95,96]. Several prospective trials have been conducted since then, comparing RIC allo-SCT with single or tandem ASCT, that varied widely in the conditioning regimens, and showed PFS and OS ranging between 31–39 months and 35–50 months, respectively. TRM was 11–16%, aGVHD 32–40%, and cGVHD up to 66% [97,98,99,100]. Hence, the oscillation has been towards reduced-intensity conditioning with regimens that incorporate intermediate doses of active anti-multiple myeloma therapy. The most popular approach in the United States is a combination of fludarabine and melphalan at a dose of 140 mg/m^2^. A CIBMTR analysis in 2011 showed steadily improving outcomes with allo-SCT due to decline in TRM, which is probably related to the increase in the use of RIC conditioning [101]. No maintenance therapy was given after the allo-SCT. Prior studies have suggested that allo-SCT may overcome the adverse effect of high-risk cytogenetics such as t(4;14) or del17p [102,103,104]. Long term follow-up of DSMM V study, which was a prospective phase 3 trial comparing ASCT followed by RIC allo-SCT to tandem ASCT in NDMM with del13q, showed that PFS favored allo-SCT group with no difference in OS [105]. Median PFS with auto/allo was 34.5 months versus 21.8 months with tandem ASCT (*p* = 0.003; adjusted hazard ratio 0.55, 95% confidence interval 0.36–0.84). Median OS was 70.2 (auto/allo) versus 71.8 months (tandem ASCT) (*p* = 0.856). The lack of overall survival benefit was due to the increased NRM from the toxicity of allo-SCT. Two-year NRM was higher with auto/allo (14.3% versus 4.1%; *p* = 0.008). In patients with both del13q and del17p, median PFS and OS, respectively, were 37.5 and 61.5 months with auto/allo (*n* = 19) versus 6.1 and 23.4 months with tandem ASCT (*n* = 6) (*p* = 0.0002 and 0.032). Though the patient numbers were small, the study provided support for the use of allo-SCT in eligible patients with high-risk cytogenetics. A pooled analysis of the long-term survival of four randomized trials (Italian, Spanish PETHEMA, and EBMT-NMAM2000 and BMT-CTN studies) comparing auto-auto vs. auto–allo after induction therapy based on availability of HLA-matched sibling donors in NDMM patients was recently published [106]. Patients received auto-auto (*n* = 899) or auto-allo (matched-sibling) (*n* = 439). Median follow up of survivors was 118.5 months. Median OS was significantly better in the auto-allo group—78.0 months in auto-auto and 98.3 months in auto-allo (HR = 0.84, *p* = 0.02). OS was 36.4% vs. 44.1% at 10 years (*p* = 0.01) for auto–auto and auto–allo, respectively. PFS was also better in auto-allo (HR = 0.84, *p* = 0.004). However, the risk of NRM was higher in auto–allo (10 year 8.3% vs. 19.7%, *p* < 0.001), while risk of disease progression was higher in auto–auto (10 year 77.2% vs. 61.6%, *p* < 0.001). Median post relapse survival was 41.5 months in auto–auto and 62.3 months in auto–allo (HR = 0.71, *p* < 0.001), supporting the role of durable graft versus myeloma (GVM) effect improving the efficacy of salvage therapies.

Allo-SCT in the relapsed setting may offer long-term PFS and OS, especially in young patients with high-risk disease and in those who relapsed within 18 months from initial ASCT [107,108,109]. These studies show that PFS and OS can be prolonged by 7–10 years in 24–31% of relapsed patients who receive RIC allo-SCT, with reduced TRM and GVHD.

Allo-SCT is traditionally performed with use of HLA-identical siblings or matched unrelated donors. Since such donors are not always available, allo-SCT from haploidentical related donors has been developed and increasingly used. Post-transplant cyclophosphamide (PT-Cy) has been used to selectively deplete allo-reactive T cells in haploidentical-SCT [110]. High-dose cyclophosphamide has been successfully used to prevent GVHD in unrelated, HLA-matched sibling and haploidentical bone marrow/PBSC transplants in various studies due to its immunosuppressive effects [111,112]. PT-Cy administered early post HSCT preferentially kills allo-reactive T cells while sparing resting, non-allo-reactive T cells, leading to suppression of GVHD as well as graft rejection [113]. In a single institution study of MM patients undergoing allo-SCT conducted by Donato et al., cGVHD had a favorable impact on OS and PFS [114]. Further research is needed to reduce the incidence of both acute and chronic GVHD and refine the conditioning therapy regimens to lower the NRM further before allo-SCT can be adopted into routine clinical practice. Trials are underway to investigate the role of novel agents as part of conditioning regimens and maintenance therapy following allo-SCT to improve the survival and long-term outcomes. Given the rapidly evolving treatment scenario and promising findings, allo-SCT holds the potential for better survival outcomes for a selected MM population that have high-risk disease with a poor long-term prognosis.

Unlike in the upfront setting, the role of allo-SCT in the treatment of RRMM patients has not been extensively studied. The following guidelines have been proposed by the International Myeloma Working Group together with the Blood and Marrow Transplant Clinical Trials Network, American Society of Blood and Marrow Transplantation, and the European Society of Blood and Marrow Transplantation committee. (1) Allogeneic HCT should be considered appropriate therapy for any eligible patient with early relapse (less than 24 months) after primary therapy that included an ASCT and/or high-risk features (i.e., cytogenetics, extramedullary disease, plasma cell leukemia, or high lactate de-hydrogenase); (2) allogeneic HCT should be performed in the context of a clinical trial if possible; (3) the role of post-allogeneic HCT maintenance therapy needs to be explored in the context of well-designed prospective trials; and (4) prospective randomized trials need to be performed to define the role of salvage allogeneic HCT in patients with MM relapsing after primary therapy [115].

### 4.7. Role of MRD in Transplant

ASCT in MM not only prolongs remission but also yields deeper responses, as was seen in the IFM/DFCI 2009 study. Patients in the transplant arm had higher MRD negativity rate compared to those in the non-transplant arm (29.8% vs. 20.4%, *p* = 0.01). It has been shown in several studies so far that MRD negativity is associated with longer PFS and OS, and if sustained, the first step towards functional cure for MM. The effect of MRD negativity on the outcomes is agnostic of the treatment regimen used or the method of determination. This provides a rationale to test the hypothesis that MRD testing could be used to determine the timing of ASCT in the upfront management of MM. Patients who fail to achieve MRD negativity after a fixed number of cycles of induction therapy can be taken for ASCT, while in those who achieve MRD negativity, transplant could be delayed with ongoing surveillance for resurgence of disease. Several ongoing clinical trials in the setting of post-SCT maintenance are incorporating MRD as a primary end-point to determine the optimal duration and intensity of therapy [116].

## 5. Conclusions

Since the 1980s, ASCT has played a significant role in the treatment of multiple myeloma. It has become a standard treatment as a consolidation therapy in newly diagnosed MM and as salvage therapy in relapsed MM. The institution of the novel agents such as immune modulators and proteasome inhibitors as first line therapy has further improved the PFS and OS of MM patients. The addition of post-ASCT maintenance therapy has even further improved the median PFS from 23–27 months to 47–53 months and the median OS from 3–5 years to 7–10 years. The addition of anti CD38 monoclonal antibodies as part of first-line therapy is showing further improvement in short-term PFS and deepening of response to MRD negative status, with the goal of further prolonging PFS and OS. The addition of a second agent to the standard single agent maintenance therapy post ASCT with the goal to further improve on the PFS is underway (SWOG1803). Other methods to improve on PFS and OS, such as chimeric antigen T-cells (CAR-T) as consolidation post ASCT in high risk patients, CAR-T in patients who fail to achieve very-good partial response or better after at least six months of maintenance post ASCT, and dendritic cell vaccine post ASCT (BMT/CTN 1401), are in protocol stages, accruing and/or completed and awaiting results. Allo-SCT plays a critical role in high-risk patients, especially young patients who relapse within 18–24 months from initial ASCT. Methods to reduce GVHD and TRM are improving to allow allo-SCT to be utilized early in the disease course. However, despite the progress made so far, the problem of chemoresistant disease persists requiring the development of agents with novel mechanism of action for RRMM. Bispecific antibodies (BsAbs) are another immunotherapy option, whereby tumor cell death can be achieved by an enhanced interaction between immune cells (T cells) and tumor cells, with a potential role in the management of RRMM [117,118]. Several immune targets including but not limited to B-cell maturation antigen (BCMA), CD38, CD138, G-protein coupled receptor family C group 5 member D (GBR1342), and CD19 are currently being tested in pre-clinical/clinical studies. In addition, preclinical studies are underway testing BsAbs enagaging natural killer (NK) cells [119]. In summary, a combination of novel immunotherapeutic agents and auto-ASCT appears to be a promising strategy for MM patients in the future.

## Figures and Tables

**Table 1:** Randomized studies comparing ASCT with conventional chemotherapy as consolidation therapy.

Study	Induction	ASCT/Chemo Regimen	Post-SCT Maintenance	PFS	OS
IFM 90 [6]	4–6 alternating cycles of VMCP/BVAP	Mel 140 + TBI vs. total 18 cycles of VMCP/BVAP	Interferon-alfa	Median EFS: 27 mo (ASCT) vs. 18 mo (chemo) *p* = 0.01	5-year OS: 52% (ASCT) vs. 12% (chemo) *p* = 0.03
SWOG 9321 [51]	4 cycles of VAD	Mel 140 + TBI vs. VBMCP for response reaches a plateau or progression	Interferon for 4 yrs vs. observation	7-yr EFS: 17% (ASCT) vs. 14% (chemo) *p* = 0.16	7-yr OS: 38% (ASCT) vs. 39% (Chemo) *p* = 0.78
MRC VII [52]	Intensive therapy (VCAP) vs. Standard therapy (BCAM)	Mel 200 or Mel 140+TBI vs. BCAM up to 12 cycles Stem cell mob with HD CTX	Interferon	Median PFS: 31.6 mo (ASCT) vs. 19.6 mo (chemo) *p* ≤ 0.001	Median OS: 54.1 mo (ASCT) vs. 42.3 mo (Chemo) *p* = 0.04
GIMEMA RV-209 [53]	4 cycles of Rd	Tandem ASCT with Mel 200 vs. MPR × 6	Randomization to R (Len) vs. observation in each arm	Median PFS: 43 mo (ASCT) vs. 22.4 mo (chemo) *p* < 0.001	4-yr OS: 81.6% (ASCT) vs. 65.3% (chemo) *p* = 0.02
RV-MM-EMN-441 [54]	4 cycles of Rd	Single or tandem ASCT vs. CRD × 6	Randomization to R (Len) or R plus prednisone until progression in each arm	Median PFS: 43.3 mo (Mel200) vs. 28.6 mo (CRD) *p* < 0.001	4-yr OS: 86% (Mel200) vs. 73% (CRD) *p* = 0.004
IFM/DFCI 2009 [55]	3 cycles of RVd	RVd × 2 following ASCT vs. RVd × 5	Len maintenance in both arms until progression (US) or for 1 year (France)	Median PFS: 47.3 mo (ASCT) vs. 35 mo (chemo) *p* < 0.001	8-yr OS: 62.2%% (ASCT) vs. 60.2%% (chemo) *p* = 0.81
EMN02/HO95 [56]	3–4 cycles of VCd	R1: Mel 200 ASCT (single or double) vs. VMP R2: VRd × 2 or no consolidation	Len maintenance for both arms until progression	3-yr PFS: 66% (ASCT) vs. 57.5% (VMP)	NR

ASCT: Autologous Stem Cell Transplantation; CRD: cyclophosphamide, revlimid, and dexamethasone; EFS: event-free survival; Len: lenalidomide; NR: not reported; MEL 140: melphalan at a dose of 140 mg/m^2^; mo: months; MPR: melphalan, prednisone, lenalidomide; OS: overall survival; PFS: progression-free survival; Rd: lenalidomide, dexamethasone; SCT: Stem Cell Transplantation; TBI: total body irradiation; VCAP: vincristine, cyclophosphamide, adriamycin and prednisolone; VCd: bortezomib (V)/Cyclophosphamide (C)/dexamethasone (D); VMCP/BVAP: alternating vincristine, melphalan, cyclophosphamide, prednisone/carmustine, vincristine, adriamycin, and prednisone; VMP: bortezomib, melphalan and prednisone; VRd: bortezomib (V), lenalidomide (R), and dexamethasone (D); yr: years.

**Table 2:** Summary of randomized controlled trials comparing single versus tandem ASCT in myeloma.

Study	First ASCT	Second ASCT	Maintenance	PFS	OS	Salvage ASCT at Relapse
EMN/H095 [56]	MEL100	None vs. Mel100	Len in both arms until progression	3-yr PFS: 73% (tandem) vs.60% single *p* = 0.03	3-yr OS: 89% (tandem) vs. 85% (single)	NR
IFM94 [58]	MEL140 + TBI (single ASCT arm) vs. MEL140 (tandem arm)	None vs. Mel 140 + TBI	Interferon α	Median PFS: 25 mo (single) vs. 30 mo (tandem) *p* = 0.03	Median OS: 48 mo(single) vs. 58 mo (tandem) *p* = 0.01	22% (single arm) vs. 28% (tandem arm)
BOLOGNA 96 [61]	MEL200	None vs. Mel120 + busulfan	Interferon α	Median PFS: 23 mo (single) vs. 25 mo (tandem) *p* = 0.001	7-yr OS: 46% (single) vs. 43% (tandem) *p* = 0.9	33% (single) vs. 10% (tandem)
GMMG HD2 [62]	MEL200	None vs. Mel200 in tandem arm	Interferon α	Median PFS: 25 mo (single) vs. 28.7 mo (tandem) *p* = 0.53	Median OS: 73 mo (single) vs. 75.3 mo (tandem) *p* = 0.33	26% (single) vs. 10% (tandem)
BMT CTN 0702 [63]	MEL200	None vs. VRd × 4 vs. tandem with MEL 200	Len until progression in all 3 arms	38-mo PFS: 58.5%-tandem 57.8%-single ASCT→VRd consolidation 53.9%-single ASCT	OS: Tandem—81.8% Single ASCT followed by VRd consolidation—85.4% Single ASCT—83.7%	NR

**Table 3:** Randomized studies done in post-ASCT maintenance therapy.

Study	Induction Therapy	Drug and Dosage of Maintenance	Duration of Maintenance	PFS/EFS (Maintenance vs. Observation)	OS (Maintenance vs. Observation)
GIMEMA RV-209 [53]	4 cycles of Rd followed by either tandem ASCT(Mel200) or MPR	Len 10 mg (3 weeks on, 1 week off)	Until progression	Cumulative median PFS: 42 vs. 22 mo (*p* < 0.001)	3-yr OS: 88% vs. 79% (*p* = 0.14)
MRC IX [78]	Up to 6 cycles of CTD or CVAD	Thal 50 mg daily for 4 weeks, increased to 100 mg daily if tolerated	Until progression	Median PFS: 22 mo vs. 15 mo (*p* < 0.0001)	Median OS: 60 mo in both arms (*p* = 0.70)
IFM 2005-02 [79]	VAD, Bort/dex Induction intensification with DCEP in 25%	Len 10 mg continuous, can increase to 15 mg preceded by Consolidation with Len 25 mg 3 out of 4 weeks for 2 cycles	Discontinued at a median time of 2 yr (range 1-3 yr) due to concerns about SPMs	Median PFS: 46 mo vs. 24 mo (*p* < 0.001)	At a median follow-up of 77 mo: 82 mo vs. 81 mo (*p* = 0.8)
CALGB 100104 [80]	Len, Thal, and/or Bort containing regimen	Len 10 mg continuous, increased to 15 mg after 3 months	Until progression or toxicity	Median PFS: 57.3 mo vs. 28.9 mo (*p* < 0.001)	5-yr OS: 76% vs. 64% (*p* < 0.0004)
MRC XI [28]	4 cycles of CTD or CRD or KCRD. Consolidation with VCD for < VGPR prior to ASCT if induction was CTD or CRD	Len 10 mg (3 weeks on, 1 week off) vs. obs	Until progression	After median f/u of 31 mo, Median PFS: 39 mo vs. 20 mo (*p* < 0.0001)	5-yr OS: 61.3% vs. 56.6% (*p* = 0.15)
TOURMALINE-MM3 [84]		Ixa 3 mg on d 1, 8, 15 out of 28 days (4 mg from cycle 5 if 3mg was tolerated) vs. placebo	2 yrs	Median PFS: 26.5 mo vs. 21.3 mo (*p* = 0.0023)	NR
NCT02253316 [85]	With PI+ IMiD in 85%, PI based (without IMiD) in 14%	Ixa 4 mg (d 1, 8, 15 out of 28-days) vs. Len 10 mg (daily for 3 mo) followed by 15 mg (daily from 4 mo onwards)	Until progression	30% on Ixa arm and 12% on Len arm progressed at a median f/u of 11.2 and 12.3 mo respectively	NR
FORTE (NCT03224507) [86]	KCdx4→ASCT→KCdx4 KRdx4→ASCT→KRdx4 KCd→stem cell collection→KRdx8	Len vs. Len +Car	Until progression	Ongoing	Ongoing
